# Book Reviews

**Published:** 1896-01

**Authors:** 


### *BOOK REVIEWS.*

Materia Medica and Therapeutics. A Practical Treatise with Especial Reference to the Clinical Application of Drugs. By John V. Shoemaker, A.M., M.D., LL.D., Professor of Materia Medica, Pharmacology, Therapeutics, and Clinical Medicine, and Clinical Professor of Diseases of the Skin in the Medico-Chirurgical College of Philadelphia. Third Edition, Thoroughly Revised. Extra Cloth, $5.00 net; Sheep, $5.75 net. Philadelphia. The F. A. Davis Co., Publishers, 1914 and 1916 Cherry street.

The third edition of this work appears in one volume, instead of two, as heretofore. In this edition the author gives place to the majority of the new remedies that have been discovered since the last edition, as well as mentioning the newer applications of old ones. Increased experience with animal extracts and juices, immunized serum and antitoxins has required that the sections devoted to discussion of these subjects be entirely rewritten in order to give a fair presentation of the present state of knowledge concerning them. The author devotes considerable space to a consideration of the therapeutic aspects of natural forces and physiological agencies, such as electricity, massage and rest cure, oxygen inhalation, medicated vapors, baths, mineral springs, and hydrotherapy, climatotherapy, diet, hypnotism and suggestion, heat and cold, etc. The chapters on electricity are especially good—clear, but complete and conservative. The preparations have been made to conform to the United States Pharmacopeia of 1890.

The present edition is a vast improvement on the preceding, and doubtless it will prove to be a very satisfactory work for both students and practitioners.

---

Supplement to the International Encyclopædia of Surgery: Edited by John Ashhurst, Jr., M.D., LL.D., Philadelphia. One royal octavo volume, of 1,136 pages, illustrated by numerous wood-engravings and a chromo-lithographic plate. Cloth, $7.50; leather, $8.50. To subscribers to the entire set, cloth, $6.00; leather, $7.00, and half morocco, $8.00. William Wood & Co., 43–47 E. 10th street, New York.

"The object of this supplementary volume is to furnish to the readers of the International Encyclopædia of Surgery a brief but sufficient account of such additions to surgical science and art as have been brought forward during the seven years which have elapsed since the revised edition of the original work was published." It seeks not to be an "ephemeris of theoretic novelties, but rather to be a trustworthy digest of accepted and established facts." It aims to go over the entire field of surgery and to bring down to date the numerous topics which were so well handled in the original volumes. This volume represents the combined work of forty-eight distinguished American surgeons and teachers, whose names are sufficient to guarantee the quality of their work. All the articles have been prepared with great care, some necessarily being more elaborate than others. The articles on the Surgery of the Head, Spine, Bones and Abdomen show, of course, the greatest amount of revision. Those who have the original Encyclopædia will need this volume to complete the series; those who have not the original set will find this volume to contain about all that is valuable among recent surgical ideas and procedures. The series as completed by this supplementary volume will long remain a standard authority among English works on surgery.

---

THE PRINCIPLES AND PRACTICE OF MEDICINE. For the Use of Practitioners and Students of Medicine. By William Osler, M.D., Professor of Medicine in Johns Hopkins University, etc. Second Edition. D. Appleton & Co., 72 Fifth avenue, New York.

The first edition of this excellent work on Practice became so popular with the profession in a short time that little need be said of this edition except to note the changes which have been made in the revision. The article on Typhoid Fever has been revised to date, and that on Malaria has been largely rewritten. The chapter on Diphtheria has been entirely recast and made to include nine additional pages. This chapter, of course, deals with the antitoxin treatment, which the author considers of great value. Much new matter has been added under Cholera, Syphilis, Tuberculosis, and other infectious diseases. The subject of Appendicitis has been rewritten and extended. Important changes and additions also are noticed under Anemia, Leukemia, Addison's Disease, Exophthalmic Goitre, and Myxedema. The section on the Nervous System contains some new matter and new illustrations. With the above changes Professor Osler has presented a work on Practice which certainly leaves little to be desired. The popularity so well earned by the first edition can only be enhanced by the second.

---

MANUAL OF GYNECOLOGY. By Henry T. Byford, M.D., Chicago. P. Blakiston, Son & Co., 1012 Walnut St., Philadelphia.

During the past few years there has been an abundant supply of works on diseases of women. But the largest part of these works have been more or less elaborate treatises suited only to the needs of the experienced practitioner and the specialist. The work before us belongs in an entirely different class. It is eminently adapted to the use of the student. It contains elementary principles without discussion of theories, and presents the subject as one having authority. It does not throw upon the reader the burden of separating the wheat from the chaff, but simply presents the wheat from which the chaff has been winnowed. The author says in his preface that he has endeavored "to supply the student with a manual complete enough for study or reference in his college course, as well as a guide to him during his first years of practice. We know of no book in which the author has more successfully accomplished the object of his endeavor. V. O. H.

---

SPECTACLES AND EYE GLASSES: Their Forms, Mounting and Proper Adjustment. By R. J. Phillips, M. D., Adjunct Professor of Diseases of the Eye, Philadelphia Polyclinic and College for Graduates in Medicine, etc. P. Blakiston, Son & Co., 1012 Walnut St., Philadelphia.

The second edition of this little book of 101 pages has just been issued. It is practical throughout, and therefore for its intrinsic worth is valuable. It contains a great deal of information that is of the utmost practical value, but such as should be familiar to every opthalmologist if he pretends at all to be familiar with his subject. The book is of far more practical value to the optician than to the oculist, except to such as are practicing their specialty away from cities where practical opticians cannot be found. It is equally true that no conscientious opthalmologist should leave the fitting and proper adjustment of spectacles entirely to the optician, but should himself see that the work has been properly done by the optician. The cuts contained in this little book are excellent, and some of the tables will be found very handy for ready reference. R.

---

THE ART OF COMPOUNDING. A Text-Book for Students, and a Reference-Book for Pharmacists. By William L. Scoville, Ph.G., Professor of Pharmacy in the Massachusetts College of Pharmacy. Price $2.50. Published by P. Blakiston, Son & Co., 1012 Walnut Street, Philadelphia.

This work was written, the author says, with a view to furnish such notes as would enable students properly to classify prescriptions, and to teach the details of pharmaceutical manipulation. The author endeavors to enunciate and classify the principles underlying each subject, and to illustrate and detail these principles so as to show their range and appreciation and to make them of practical utility at the prescription counter. The subjects treated include all the forms of administering drugs; pills, emulsions, mixtuies, powders, suppositories, ointments, etc. Under each heading there are numerous illustrative prescriptions, with suggestions as to the proper mode of compounding them. The chapter on Incompatibility is complete and practical. No doubt the book is one of the best that can be had on this subject.

---

A MANUAL OF SYPHILIS AND VENEREAL DISEASES. By James Nevins Hyde, A.M., M.D., Professor of Skin and Venereal Diseases, Rush Medical College, Chicago, etc., and Frank H. Montgomery, M.D., Lecturer on Dermatology and Genito-Urinary Diseases, etc., Rush Medical College, etc., Chicago. With forty-four illustrations in the text and eight full page colored plates. Philadelphia. W. B. Saunders. 1895. Price $2.50 net.

This is a manual of convenient size, but of sufficiently full text. The entire subject of Syphilis is well covered. Then Chancroid is taken up. Next is a description of genital lesions not purely venereal. The inclusion of these lesions in the book is especially to be commended. Venereal hypochondriasis is aptly discussed. Then come Acute-Urethritis and complications, Chronic Urethritis, and Strictures, finally Gonorrhœa in Women. Illustrations are good. The work is one which would not be out of place in the library of any student or physician. M. B. H.

---

HANDBOOK OF THE DIAGNOSIS AND TREATMENT OF SKIN DISEASES. By Arthur Van Harlingen, Ph.B., M.D., Emeritus Professor of Dermatology in the Philadelphia Polyclinic; Dermatologist to the Howard Hospital. Third Edition, revised and enlarged. Sixty Illustrations, some in colors. Philadelphia. P. Blakiston, Son & Co., 1895. Price $2.75.

Dr. Van Harlingen is a well known dermatologist of Philadelphia. This is an up-to-date treatise, in manual form, on Diseases of the Skin, written with especial reference to the wants of the general practitioner, with foot notes showing where further information upon a subject may be had.

The diseases are described in alphabetical order, instead of according to an indefinite classification. We have never seen better illustrations. It is a very desirable work. M. B. H.

---

INTERNATIONAL MEDICAL ANNUAL FOR 1896.

E. B. Treat, publisher, New York, has in press for early publication the 1896 *International Medical Annual*, being the fourteenth yearly issue of this eminently useful work. Since the first issue of this one-volume reference work, each year has witnessed marked improvements; and the prospectus of the forthcoming volume gives promise that it will surpass any of its predecessors. It will be the conjoint authorship of forty distinguished specialists, selected from the most eminent physicians and surgeons of America, England and the continent. It will contain reports of the progress of Medical Science at home and abroad, together with a large number of original articles and reviews on subjects with which the several authors are especially associated. This book is a useful reference-work for the doctor's library. The price, as heretofore, will be $2.75. Ready soon.

